# “Nourish to Flourish”: complementary feeding for a healthy infant gut microbiome—a non-randomised pilot feasibility study

**DOI:** 10.1186/s40814-022-01059-3

**Published:** 2022-05-18

**Authors:** Amy L. Lovell, Hannah Eriksen, Starin McKeen, Jane Mullaney, Wayne Young, Karl Fraser, Eric Altermann, Olivier Gasser, Martin Kussmann, Nicole C. Roy, Warren C. McNabb, Clare R. Wall

**Affiliations:** 1 Department of Nutrition and Dietetics, The University of Auckland Faculty of Medical and Health Sciences, Private Bag 92019, Auckland, 1142 New Zealand; 2 High-Value Nutrition National Science Challenge, Auckland, New Zealand; 3 AgResearch Ltd. Grasslands Research Centre, Private Bag 11008, Palmerston North, 4442 New Zealand; 4 Riddet Institute, Massey University, Private Bag 11222, Palmerston North, 4442 New Zealand; 5 Malaghan Institute of Medical Research, PO Box 7060, Newtown, Wellington 6242 New Zealand; 6 Nuritas Ltd., Dublin, Ireland; 7 University of Otago, PO Box 56, Dunedin, 9054 New Zealand

**Keywords:** Infant, Complementary feeding, Microbiome, Immune health, Feasibility

## Abstract

**Background:**

The introduction of complementary foods and changes in milk feeding result in modifications to gastrointestinal function. The interplay between indigestible carbohydrates, host physiology, and microbiome, and immune system development are areas of intense research relevant to early and later-life health.

**Methods:**

This 6-month prospective non-randomised feasibility study was conducted in Auckland, New Zealand (NZ), in January 2018. Forty parents/caregivers and their infants were enrolled, with 30 infants allocated to receive a prebiotic NZ kūmara (flesh and skin; a type of sweet potato) prepared as a freeze-dried powder, and ten infants allocated to receive a commercially available probiotic control known to show relevant immune benefits (10^9^ CFU Bifidobacterium lactis BB-12®). The primary outcome was the study feasibility measures which are reported here.

**Results:**

Recruitment, participant retention, and data collection met feasibility targets. Some limitations to biological sample collection were encountered, with difficulties in obtaining sufficient plasma sample volumes for the proposed immune parameter analyses. Acceptability of the kūmara powder was met with no reported adverse events.

**Conclusion:**

This study indicates that recruiting infants before introducing complementary foods is feasible, with acceptable adherence to the food-based intervention. These results will inform the protocol of a full-scale randomised controlled trial (RCT) with adjustments to the collection of biological samples to examine the effect of a prebiotic food on the prevalence of respiratory tract infections during infancy.

Trial registration

Australia New Zealand Clinical Trials Registry ACTRN12618000157279. Prospectively registered on 02/01/2018.

**Supplementary Information:**

The online version contains supplementary material available at 10.1186/s40814-022-01059-3.

## Key messages regarding feasibility

- What uncertainties existed regarding the feasibility existed prior to this study?

Uncertainties that required clarification before proceeding to a full-scale RCT included recruitment, recruitment rate, retention, and adequate and viable biological sample collection. Therefore, we examined the recruitment process and rate of recruitment, adherence to a food-based intervention on introduction to complementary foods, and whether participants experienced adverse events. In addition, we examined the validity of data collection methods, assessment tools used, and accuracy of parent-reported illness in this cohort.

- What are the key findings on feasibility from this study?

The recruitment response indicated that it was feasible to recruit infants (and their parents) before starting complementary feeding; however, the recruitment time was longer than anticipated. Participant retention until study completion and adherence to the daily food-based intervention were high, at 80% and 88%, CF3 and CF6, respectively. Tools for data collection (e.g. illness records, record-assisted 24-h recalls) performed well. Parental reporting of total illness events in 6–12-month-old infants was a valid data collection method. However, the ability to rely on parent-reporting of airway-related illness, including upper respiratory tract infections (URTI), decreased after 9 months of infant age, necessitating continued physician-verified illness reporting. Identification of redundant sample collection and analyses for urine and saliva and the remaining sample collection methods were adequate. Procedures for blood collection have been adjusted to improve success.

- What are the implications of the findings on the design of the main study?

It is feasible to conduct a full-scale RCT of a food-based intervention in infants starting complementary feeding, with some adaptations to the feasibility study model. The food-based intervention (kūmara powder) was acceptable for inclusion in an infant’s dietary intake without displacing other foods, and no adverse events were recorded. Biological sample collection protocols have been amended and no longer include saliva and urine collection. Improvements in the collection protocol for blood samples have been made. Strategies for streamlining parent-reported adherence, health, and dietary intake data have been implemented, developing a study-specific data collection app for iOS and Android.

## Background and rationale

Weaning is a period of marked physiological change. The introduction of complementary foods and changes in milk consumption are accompanied by significant gastrointestinal tract (GIT), immune, and developmental adaptations. Recent discoveries have highlighted how the infant immune system co-evolves with the GIT and its microbiota in a mutualistic relationship [1–3], and comparative metagenomic studies have underscored the dominant role of diet in shaping the composition and function of the GIT microbiota [4, 5].

The period of complementary feeding provides an opportunity to examine the ability of certain complementary foods and/or their products to promote immune health through changing the GIT microbiome. A reverse metabolic approach was used to inform the most promising prebiotic combinations shown to support the growth of beneficial bacteria in the infant gut [6]. From a list of twenty possible foods, kūmara (a NZ sweet potato) was chosen as the potential prebiotic intervention food as it can be safely introduced to infants during weaning.

Kūmara is widely used as an ingredient in commercial infant foods in New Zealand [7] and reconstituted food products are commonly used during weaning (e.g. infant rice cereal). Kūmara contains indigestible carbohydrates, including prebiotic resistant starch (RS), which are proposed to influence microbial communities and the maturation/immunity of the GIT.

### Study aims

This study aimed to determine the feasibility and acceptability of introducing kūmara as the first food that could be consumed daily for 6 months and promote favourable changes to the infant microbiome. The secondary aim was to determine the power of a large-scale RCT to investigate the efficacy of kūmara with increased RS in reducing episodes of upper respiratory tract infections (URTIs) in infants from 6 to 10 months of age.

### Study objectives

This feasibility study had the following objectives:

- To evaluate the feasibility of the study processes, including recruitment rate, retention rate, study completion, and data collection
- To evaluate the processes and acceptability of collecting and analysing biological samples (breastmilk, stool, blood, and urine)
- To validate the self-reported health event diary with physician-confirmed health events
- To determine adherence to the kūmara intervention, assessed as the daily consumption of 5 g of kūmara powder reconstituted with water to approximately 1 tsp kūmara paste
- To determine whether the volume of kūmara consumed resulted in changes to the GIT microbiome and associated metabolites
- To identify the types of potential adverse events
- To determine the number of infants required for an RCT of a complementary feeding intervention for improved immune protection from infection

## Methods

### Study design

**Fig. 1:**
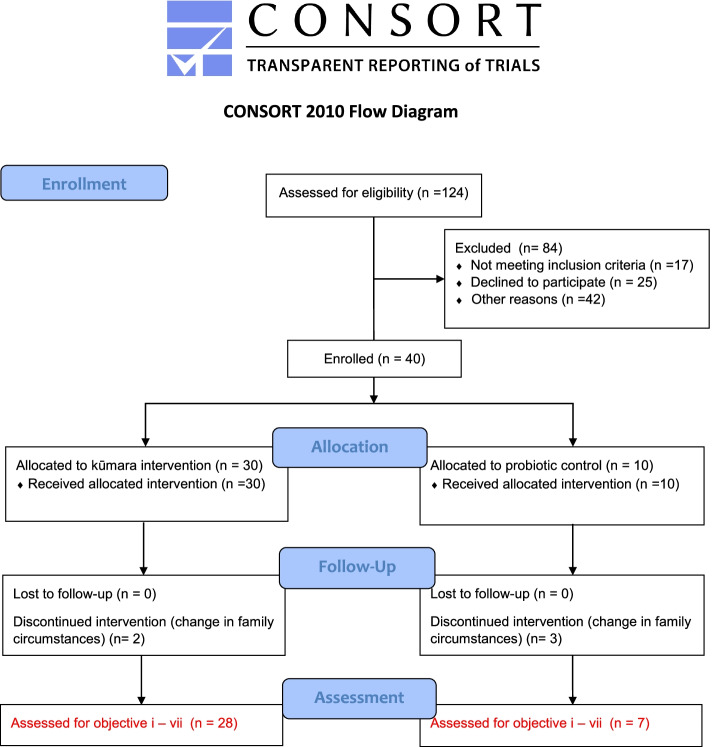
Trial outline of 40 infants participating in the “Nourish to Flourish” infant complementary feeding pilot feasibility study (2018–2019)

“Nourish to Flourish” was designed as a single-centre, non-randomised pilot feasibility study. Consenting mothers of 40 healthy infants received a dietary intervention based on a prebiotic NZ kūmara (including flesh and skin) prepared as a freeze-dried powder (*n* = 30) or commercially available probiotic control (*n* = 10) known to show relevant immune benefits (10^9^ CFU Bifidobacterium lactis BB-12®) [8–11] The probiotic intervention served as a positive control to ensure that our design and methods, in principle, can detect probiotic-related immune benefits. The CONSORT extension for pilot and feasibility trials flow diagram [12] is shown in Fig. 1. Data collection occurred at three time points, baseline (infant age < 6 months), 3 months (infant age 8–9 months), and 6 months (infant age 12 months). The protocol was prospectively registered with the Australian NZ Clinical Trials Registry, reference: ACTRN12618000157279.

The study was conducted per the Declaration of Helsinki and was approved by the NZ Health and Disability Ethics Committee (HDEC), reference 17/NTA/239. Written informed consent was obtained from parents on the infants’ behalf. All data were collected according to the Good Clinical Practice and quality control procedures.

### Study setting

The study took place at the University of Auckland Clinical Research Centre.

### Recruitment of participants

The target population for recruitment was healthy and appropriate for gestational age infants < 6 months of age. Recruitment occurred from January to August 2018 via social media outreach. It was expected that enrolled infants would be introduced to complementary foods at around 6 months, as per the NZ Ministry of Health Guidelines [13]. Infants were not eligible for this study if they were born small for gestational age (SGA) or preterm, had a health condition that affected feeding, or were diagnosed with a digestive or metabolic disorder. Infants were enrolled in the study before the introduction of complementary foods (i.e. < 6 months of age) and commenced the intervention on the introduction of complementary foods at around 6 months of age, as per the Dietary Guidelines [13].

### Intervention

The dietary intervention and probiotic were supplied to participants at no cost. Parents were requested to introduce the kūmara powder (provided as a daily dose in a single 5 g sachet) or probiotic daily for 6 months (from when their infant started complementary feeding until they turned 12 months of age). Preparation instructions were to reconstitute the kūmara powder with water to an age-and-stage appropriate consistency, which could be eaten on its own or combined with foods. The amount given to the infant (approx. 1 tsp) provided the nutrient equivalent (including RS) of a 25-g piece of kūmara however did not displace other foods in the infant’s diet. Adherence was measured using a daily record of intervention consumption where parents were asked to prepare a single sachet of kūmara powder as per instructions and record the proportion of kūmara paste consumed, e.g. 50% = half a sachet. Adherence was defined as the number of days where 100% of kūmara was consumed in the previous month.

The positive control provided a known amount of a specific probiotic (10^9^ CFU Bifidobacterium lactis BB-12®) that has been shown to change the microbiota in a certain way [14, 15] to standardise the microbiota in the control group taking into consideration the variation in dietary intake that can occur during weaning.

### Data collection and measurements

Infants attended clinic appointments at enrolment (CF1) when the infant was < 6 months of age and 3 (CF3) and 6 months (CF6) later. Daily illness records were completed prospectively by parents, reporting illness incidence, illness duration, assessment by a medical professional, and medications prescribed or administered. The research team checked these records for completeness at each visit, asking questions for clarity where needed.

#### Demographics

Parent sociodemographic information was collected at baseline using electronic questionnaires generated using the Qualtrics© software (Qualtrics, Provo, UT).

#### Illness questionnaire

Health-related events were classified into four groups: (1) airway-related illness, (2) skin-related illness, (3) childhood infections, and (4) other illnesses. The incidence of airway-related diseases, particularly respiratory tract infections (RTIs), and the ability of parents to correctly identify these were of interest to the study. In NZ, the primary physician has a central position in the healthcare service, keeping records on every enrolled patient through a unique National Health Index number (NHI), making them a reliable external data source [16, 17]. Each infant’s primary physician was recorded on enrolment, and written informed consent to contact the nominated physician to verify health information was obtained. Infection-related visits, medication prescriptions, hospitalisations, and other illnesses during the intervention period were obtained and reviewed by a trained member of the research team and compared with parent-reported health diaries. The same person reviewed all the records. When an event was recorded by the primary physician only, it was coded as a “missed event”.

#### Anthropometry

Weight (kg) and length (cm) were taken at CF1, CF3, and CF6 according to the standardised protocols [18].

#### Dietary intake

Dietary intake was measured at CF3 and CF6 using 3-day food records. The food records were entered into a food composition software programme FoodWorks 10 Professional, v10.0. (Brisbane: Xyris Pty Ltd., 2019) to determine the nutritional composition of foods and nutrient intake at each time point. Three-day food records were chosen as the assessment method to capture the complexities and diversity in dietary intake. Breastmilk is not quantified in the food record. Instead, a “breastmilk” entry was selected on FoodWorks, and one serving was entered for each breastfeeding occasion. Breastmilk volume was estimated according to previously validated methodology, where the quantity (mL) was estimated using feeding duration (minutes) and a conversion factor of 10 mL per minute, with a maximum of 10 min of breastfeeding or 100-mL breastmilk [19, 20]. If breastmilk was expressed/pumped and fed to a child in a bottle, the quantity (mL) consumed at each feeding was recorded [21].

### Biological samples

#### Blood

Specific consent was sought to collect two blood samples at CF1 and CF6. A trained phlebotomist collected the blood sample. A 2–5-mL sample was required for the following analyses: (1) to assess the antibody response in plasma following the oral rotavirus immunisation [22], (2) to assess the antibody response in plasma following the pneumococcal vaccination [23, 24], and (3) to isolate peripheral blood mononuclear cells (PBMC) and characterise the B cell receptor (BCR) repertoire [25]. In addition, a plasma sample was obtained for global metabolite profiling [26, 27]. Total antibody titres in the plasma at 6 and 12 months of age were used as a non-specific measure of protective antibody responses. As a secondary outcome, this study aimed to determine the feasibility of collecting a sufficient volume of blood to measure vaccine response as an indicator of immune status.

#### Urine

Samples were collected at CF1, CF3, and CF6 for global metabolite profiling. At each clinic visit, a Tegaderm® 4 × 5-in. transparent plastic dressing film was placed over the area where urine is voided in a clean nappy, along with a cotton pad to absorb the urine sample. The nappy was checked after 15–20 min to see if the cotton pad was wet. Following successful collection on the cotton pad, the pad was placed into a sterile 20-cc syringe. The syringe plunger was used to compress the pad and expel the urine sample. The sample was collected into two prelabelled cryovials and frozen at − 80°C until analysis.

#### Stool

Samples were collected at CF1, CF3, and CF6 according to a predefined protocol [28]. Samples were used to assess the extent to which complementary feeding had modified the infant GIT microbiota and quantify vaccine-specific antibody responses. Parents/caregivers were provided with stool collection kits to collect and store stool samples at home and successfully transport them into the clinic. At CF1, a CHUX® cloth was used to line a clean nappy and collect a stool sample. Instructions were given to ensure that samples were not contaminated by urine. The liner was removed following a bowel motion and rolled up into a 25-mL collection pot. At CF3 and CF6, where stool samples were more formed due to consuming complementary foods, a sterile 25-mL collection pot with a scoop was used to collect the sample. The scoops transferred approximately 1 tsp of stool into the pot. All stool samples were stored in the home freezer until the clinic visit and transported with a frozen gel pack in a polystyrene box. Once returned to the clinic, samples were labelled and frozen at − 80 °C until analysis. DNA isolation was performed using standard protocols. Shot-gun metagenomic sequencing was conducted at the APC Microbiome Institute, Teagasc, Cork, Ireland, using the Illumina NextSeq paired-end 150 bp × 2 sequencing. To determine the protective antibody response at each time point, faecal supernatants were analysed using standard ELISA protocols for human RV-specific IGA and IgG.

#### Saliva

Samples were collected to quantify vaccine-specific antibody responses at CF1, CF3, and CF6 as a non-specific measure of protective antibody responses. Samples were collected according to a predefined protocol [28]. A cotton wool swab was placed in the infant’s mouth until saturated (1 to 3 min). Compression was used to recover the sample. The saturated swab was placed in a 5-mL syringe, and the plunger was compressed to express saliva into a pre-labelled cryovial. All saliva aliquots were stored at − 80 °C until analysis. Aqueous metabolites were detected using global metabolite profiling in saliva samples. For immune analysis, samples were centrifuged at 2000*g* for 10 min, and the clarified supernatant was analysed using ELISA for human RV-specific IgA and IgG.

#### Breastmilk

Samples were collected from breastfeeding mothers where possible at CF1, CF3, and CF6. Samples were collected at home according to a standardised method [28]. Approximately 12–15 mL of breastmilk was collected into a sterile 15-mL Falcon tube. The date of collection and collection times were recorded. Samples were transported to the clinic with a frozen gel pack in a polystyrene box and processed immediately. Approximately 2 mL of the whole breastmilk sample was aliquoted into two 1.8-mL cryovials. The remaining 12 mL was centrifuged immediately at 3000*g* for 10 min, twice. The supernatant was then aliquoted into 1.8 mL cryovials and stored at − 80 °C until analysis. Breastmilk samples (breastmilk supernatant) were only used for quantification of immune factors (using ELISA), and the microbiome and metabolome were not investigated in this feasibility study.

#### Adverse events

Parents were asked to record any adverse events that could be associated with the consumption of the kūmara intervention. Adverse events included, but were not limited to, vomiting, diarrhoea, urticaria, and intolerance. Kūmara is not a common cause of IgE-mediated allergies; however, intolerances could occur. A standard operating procedure was in place for the reporting of adverse events to the research team.

### Data management

Study questionnaires were exported from Qualtrics into a password-protected Excel spreadsheet saved in a secure folder on the University of Auckland server, only accessible to the research team. All essential documents, including source documents, were stored securely. They will be retained for a minimum of 10 years following the youngest participants’ sixteenth birthday.

### Criteria for assessing study feasibility

The following criteria were used to assess the feasibility of the study: (1) minimum 80% recruitment rate, (2) minimum study completion rate of 80%, (3) collection and analyses of at least 80% of all biological samples, and (4) 80% completion rate of food and health records.

### Statistical analysis

All statistical analyses were performed using IBM SPSS Statistics for Windows, version 26.0 (IBM Corp., Armonk, NY, USA). The analyses were exploratory and were not intended to test the effectiveness of the intervention. Descriptive statistics are presented as numbers (per cent) to characterise participants enrolled in the pilot study. Data from food records were analysed using Foodworks 10 Professional, v10.0. (Brisbane: Xyris Pty Ltd., 2019) for macronutrient and micronutrient intake.

For the validation of self-report of illness, the total number of illness events for an infant (*N*) was considered as the sum of the events registered by parents (*n*) and any additional missed events found in the primary physician records (*a*), i.e. *N* = *n* + *a*. Completeness of the parent record (*C* = *n*/*N* × 100) was estimated as the number of illness events recorded (*n*) as a percentage of the total number of illness events (*N*).

## Results

A participant flow diagram shows the number of expressions of interest in the study, the number eligible for inclusion, allocated to the intervention, and included in the analysis (Fig. 1). Further detail of the data collected at each time point are presented in the supplementary material (Fig. 1).

### Recruitment, attrition, and feasibility of an infant complementary feeding intervention

Forty healthy infants < 6 months of age were recruited into this study over 6 months (February to August 2018). One hundred and twenty-three participants expressed interest, with an average of 7 participants enrolling per month. The estimated accrual period was 4 months (10 infants per month) to achieve the sample size. The rate of recruitment was 70% of expected, with recruitment taking 2 months longer than predicted. Overall, the attrition rates were low (12.5%). Three infants withdrew from the study before CF3 and two before CF6, all due to the changes in family circumstances making ongoing participation impossible. No adverse events were recorded.

### Sample collection and assessment tools

Participant characteristics and infant anthropometry are presented in Table 1. The feasibility measure aspects of the study in terms of biological sample collection, analysis, and accuracy of reporting dietary intake and health events at each time point are displayed in Table 2. Data collection was ≥ 80% at both time points for intervention adherence, questionnaire completion, anthropometry, and stool sample collection. The collection of viable stool and breastmilk samples for analyses met the feasibility targets at each time point. Blood, urine, and saliva samples did not meet the feasibility targets for analysis.

**Table 1:** Baseline characteristics of families participating in the “Nourish to Flourish” infant complementary feeding pilot study (2018–2019)

Characteristic	Kūmara powder, ***n*** = 30	Probiotic control, ***n*** = 10
Sex, *n* (%)		
Boy	15 (50)	4 (40)
Girl	15 (50)	6 (60)
Mother ethnicity, *n* (%)		
NZ European	20 (67)	9 (90)
Māori	1 (3)	0 (0)
Pacific	2 (7)	0 (0)
Asian	2 (7)	0 (0)
Others	5 (17)	1 (10)
Mother education, *n* (%)		
None	1 (3)	0 (0)
Primary	0 (0)	0 (0)
Secondary	1 (3)	0 (0)
Tertiary	23 (77)	9 (90)
Others	0 (0)	0 (0)
*Missing*	*1*	*1*
Mother work, *n* (%)		
Full time caregiver	14 (47)	9 (90)
Full time employment	5 (17)	1 (10)
Part time employment	4 (13)	0 (0)
Receiving benefit	0 (0)	0 (0)
Unemployed, no benefit	2 (7)	0 (0)
Others	5 (17)	0 (0)
Father ethnicity, *n* (%)		
NZ European	17 (57)	5 (50)
Maori	1 (3)	0 (0)
Pacific	2 (7)	0 (0)
Asian	3 (10)	0 (0)
Others	6 (20)	5 (50)
*Missing*	*1*	
Father education, *n* (%)		
None	0 (0)	0 (0)
Primary	0 (0)	0 (0)
Secondary	3 (10)	0 (0)
Tertiary	22 (73)	9 (90)
Others	4 (13)	1 (10)
*Missing*	*1*	
**Infant anthropometry**		
Weight (kg), mean ± SD		
Birth	3.5 (0.5)	3.7 (0.3)
Baseline	6.6 (1.0)	6.8 (0.7)
Length (cm), mean ± SD		
Birth	51.5 (2.9)	51.3 (1.8)
Baseline	63.2 (3.3)	64.3 (2.5)

**Table 2:** Feasibility outcomes of data collected across three time points in the “Nourish to Flourish” infant complementary feeding feasibility pilot study (2018–2019)

Completed	Baseline (*N* = 40)	CF3 (*N* = 37)	CF6 (*N* = 35)
Questionnaires—*sociodemographic characteristics, general health, birth outcomes*	40 (100)	N/A	33 (94)
Anthropometry—*weight, length*	40 (100)	37 (100)	35 (100)
Intervention adherence^a^			
Kūmara^b^	N/A	22 (80)	25 (88)
Probiotic^c^	N/A	7 (80)	5 (81)
Dietary intake			
Collected	N/A	37 (100)	33 (94)
Analysed	N/A	36 (97)	32 (97)
Health data—*parent-recorded*			
Completed	N/A	37 (100)	35 (100)
GP-verified reported illnesses—*airway-related illness*	N/A	23 (82)	4 (40)
GP-verified reported illnesses—*skin-related illness*	N/A	7 (71)	2 (50)
GP-verified reported illnesses—*gastrointestinal*	N/A	10 (60)	5 (80)
Stool—*microbiome, metagenome (LC-MS)*			
Collected	38 (95)	37 (100)	34 (97)
Analysed	36 (95)	36 (97)	33 (97)
Stool—*protective antibody response (ELISA)*			
Collected	30 (75)	31 (84)	30 (86)
Analysed	24 (80)	29 (94)	24 (80)
Saliva—*microbiome*			
Collected	40 (100)	37 (100)	34 (97)
Analysed	35 (88)	29 (78)	13 (38)
Saliva—*anti-body titres (ELISA)*			
Collected	40 (100)	37 (100)	34 (97)
Analysed	26 (65)	27 (73)	19 (56)
Breast milk—*antibody titres (ELISA)*			
Collected	39 (98)	30 (81)	19 (54)
Analysed	39 (98)	27 (90)	19 (100)
Blood (plasma)*—RVV-specific IgG quantification*			
Collected	22 (55)	N/A	11 (31)
Analysed	22 (100)	N/A	10 (91)
Urine*—metabolome*			
Collected	31 (78)	30 (81)	26 (74)
Analysed	0 (0)	0 (0)	0 (0)

Values are displayed as *n* (%) unless otherwise stated

The collected samples were determined as the number of successful sample collections from each time point. Samples analysed were determined as the number of viable samples for analysis from those collected

*Abbreviations*: *3DFR*, 3-day food record; *N/A*, not applicable at this time point

^a^Adherence is defined as consuming the intervention on 80% of the recorded days (month)

^b^Thirty infants receiving kūmara *n* = 28 at CF3 and CF6

^c^Ten infants receiving probiotic; *n* = 9 at CF3 and *n* = 6 at CF6

#### Biological sample analysis

For infants where blood was collected, the volume collected at both time points was insufficient to obtain enough PBMC to perform the BCR repertoire analysis or enough sample to extract sufficient plasma. Plasma collection was below 50% at both time points. The volume of urine samples collected was sufficient; however, all samples were contaminated with faecal matter, rendering the sample inappropriate for metabolome analysis. The collection of urine samples was therefore not recommended for the RCT.

The collection of saliva samples was ≥ 97% at each time point. However, samples were frequently contaminated with breastmilk or food residue. Twenty-one saliva samples were contaminated at CF1, 8 (22%) at CF3, and 1 (3%) at CF6, rendering them inappropriate for microbiome and metabolome analyses. Volume was also an issue, with 5 (13%) samples at CF1 being of inadequate volume for all analyses, 8 (22%) at CF3, and 22 (63%) at CF6. Saliva samples were viable for analysis of antibody titres (ELISA).

The collection of faecal samples was > 80% at each time point. At baseline, two samples were not sufficient in volume for analysis, and one sample at CF3 and CF6 could not be analysed due to a logistical error. Difficulties in extracting faecal metabolites from the CHUX® cloth were apparent at CF1 due to the absorbency of the cloth. The results of the faecal microbiome analyses demonstrated an increasing diversity of microbiota in the operational taxonomic units (OTUs) over the 6 months of the intervention. In addition, they demonstrated that each infant who had not yet started eating solid foods had a microbiome that were more like each other than older infants consuming varied diets (McKeen et al. unpublished data).

Breast milk collection decreased through the course of the trial due to the cessation of breastfeeding in eleven mother-infant pairs. Breastmilk samples were acceptable for immune analysis (Table 2). Rotavirus vaccine (RVV)-specific antibody responses showed no significant difference in RVV-specific faecal IgA levels between the groups before or after vaccination. No correlation was found between faecal and breastmilk RVV-specific IgA levels at CF1 and CF3. RVV-specific IgG quantification was only performed on available blood samples and did not show significant differences between the groups.

### Intervention adherence

Adherence (kūmara and probiotic groups) to the study protocol was > 80% at CF3 and CF6. When examined individually, adherence to the kūmara intervention was 80% at CF3 and 88% at CF6 (Table 2).

### Dietary intake

Dietary intake was recorded by 100% and 94% of participants at CF3 and CF6, respectively (Table 2). The quality of each record varied, with clarification of food quantities, types of foods, and recipes used required, particularly infants who had other adults involved in their food intake (i.e. attending daycare). Two records (one at each time point) were excluded from the dietary analysis as they were incomplete and could not be verified.

For dietary diversity analysis, foods were categorised into groups and nutrient sources. A phenetic hierarchical tree was created, adapting the methodology reported by Johnson et al. [29] within the context of infant dietary intakes. The proposed tree-based analysis had two levels manually developed by the research team. Level 1 broadly categorises the food based on food groups reflected in the Dietary Guidelines [13], and level 2 categorises foods by their composition/ingredients. For example, the levels of the tree for a commonly consumed food such as banana were denoted as L1_Fruit; L2_banana (Supplementary Material, Table 1).

### Validation of parent-reported RTI incidence

Four (11%) and 13 (37%) infants at CF3 and CF6, respectively, did not experience any illness events. Twenty-five per cent of primary physicians did not respond to requests to verify the infant’s health record at CF3 and 27% at CF6, resulting in 12 and 14 illness events from 8 and 6 infants at CF3 and CF6, respectively being unable to be verified and excluded from the validation analysis.

Eighty-three illness events and 50 other events were recorded at CF3 and CF6, respectively. Table 3 displays the completeness of all illness reporting. Twenty-three total illness events were recorded by the primary physician but were missed by parent-reporting at CF3 and 15 at CF6, resulting in classification as a “missed event”. Of these, five were URTIs at CF3 and seven at CF6. Overall agreement between parent-reporting and primary physician verification of all illness events was 76% at CF3 and 74% at CF6. Agreement for total airway-related illnesses was 82% (75% for URTIs) at CF3, decreasing to 40% (25% for URTIs) at CF6.

**Table 3:** Frequencies and completeness of illness diagnoses at CF3 and CF6 of children participating in the “Nourish to Flourish” infant complementary feeding pilot study (*N* = 40)

Diagnosis	Parent-reporting (***n***)		Health care provider record		Missed events		Total (***N***)		Completeness (***n***/***N***)	
	CF3	CF6	CF3	CF6	CF3	CF6	CF3	CF6	CF3	CF6
**Airway-related illness**	**23**	**4**	**24**	**9**	**5**	**6**	**28**	**10**	**0.82**	**0.40**
URTI	15	2	20	8	5	6	20	8	0.75	0.25
Common cold	1	2	0	1	0	0	1	2	1.00	1.00
Cough	4	0	3	0	0	0	4	0	1.00	0.00
Chest infection	1	0	0	0	0	0	1	0	1.00	0.00
Bronchiolitis	2	0	1	0	0	0	2	0	1.00	0.00
**Skin-related illness**	**5**	**1**	**7**	**2**	**2**	**1**	**7**	**2**	**0.71**	**0.50**
Urticaria	3	1	5	2	2	1	5	2	0.60	0.50
Eczema	1	0	1	0	0	0	1	0	1.00	0.00
Nappy rash	1	0	1	0	0	0	1	0	1.00	0.00
**Childhood infections**	**16**	**12**	**16**	**11**	**2**	**0**	**18**	**12**	**0.89**	**1.00**
Fever	9	9	10	9	2	0	11	9	0.82	1.00
Ear infection	7	3	6	2	0	0	7	3	1.00	1.00
**Other illness**	**31**	**23**	**37**	**26**	**14**	**7**	**45**	**30**	**0.69**	**0.77**
Diarrhoea and vomiting	6	4	6	4	1	1	7	5	0.86	0.80
Constipation	0	0	3	0	3	0	3	0	0.00	0.00
Conjunctivitis	2	3	2	4	0	1	2	4	1.00	0.75
Allergy	3	7	5	5	2	0	5	7	0.60	1.00
Oral thrush	1	3	0	3	0	0	1	3	1.00	1.00
Virus	2	1	6	2	4	1	6	2	0.33	0.50
Injury	0	0	1	0	1	0	1	0	0.00	0.00
GOR	1	0	1	1	0	1	1	1	1.00	0.00
Others (not listed)	4	2	1	4	0	2	4	4	1.00	0.50
**Total health issues**	**63**	**37**	**72**	**45**	**20**	**13**	**83**	**50**	**0.76**	**0.74**

*CF3*, complementary feeding 3 (infant age approx. 9 months); *CF6*, complementary feeding 6 (infant age approx. 12 months); *URTI*, upper respiratory tract infection; *GOR*, gastro-oesophageal reflux

## Discussion

This study sought to evaluate the feasibility of providing a food-based intervention to infants during early complementary feeding with prebiotic properties that support the growth of beneficial gut bacteria. This study also sought to determine adherence to its consumption. Secondary objectives were to determine whether viable biological samples could be collected to assess the microbiome, metabolites, and immune function. It was feasible to recruit infants before introducing complementary foods and retain them in a feeding study for six 6 months. The recruitment rate would be acceptable if the study were scaled up for the RCT if allowances for an extended recruitment period were made in the study timeline. The study also demonstrated that it was possible to feed the prebiotic food (kūmara) as required to infants every day for over 6 months with no adverse events.

Sample size calculations for a full-powered RCT are based on the prevalence of URTIs. Prevalence is defined as at least one RTI episode at four months post-randomisation. On the assumption of detecting an absolute difference of 20% between the intervention and control groups, a future RCT would need 240 infants (80% power and 5% level of significance). The total sample size would need to be 300 infants to allow for a loss to follow up of 20%.

Successful collection of health parameters and most biological samples was possible; however, not all samples were considered viable for analysis due to issues such as contamination with faeces (urine samples) or from feeding (saliva samples) or collection of an insufficient sample volume (faecal, blood, and saliva samples). Alterations in the collection and analysis protocols for stool samples were needed, particularly at CF1 where the consistency of the sample (more liquid before the introduction of complementary food) was more prone to contamination with urine in the nappy. The protocols for the isolation of microbiota from the samples were consistent at all time points; however, protocol amendments such as providing a single type of nappy liner without added chemicals, use of separation bags with buffer to remove the sample from the liner for microbiome analysis (16s rRNA), and modifying protocols (e.g. increasing the aqueous extraction solvent) to account for resorption by the liner for lipid metabolite detection were necessary for the RCT. Due to the limited range of aqueous metabolites, variation in acceptable collection methods, unpredictable feeding needs of infants, and risk of contamination, saliva samples were not recommended for the RCT.

Collecting urine samples is not common in this age range, due to issues with faecal contamination. Protocols for reducing the risk of contamination of urine samples are available, however, significantly increase participant burden through collecting samples over 4 h using adhesive plastic bags or through assessing mannitol:lactulose excretion ratios. The collection of blood samples was limited due to the difficulties in achieving adequate venous access in participating infants. The proposed secondary immune outputs in the large-scale RCT necessitate further investigation into phlebotomy and blood collection techniques in healthy, well-nourished infants, such as the use of an infra-red vein finder.

The level of agreement between parent-reporting of any illness and medical records of infants was high at both time points. However, agreement in reporting airway-related illness reduced from > 80% at CF3 to 40% at CF6. Potential contributing factors to these differences may include changing life circumstances such as returning to work, inconsistent sources of medical care, or accuracy of recall being dependent on the perceived seriousness of an event [2]. For example, three out of four mothers (75%) had returned to work during this record period, possibly impacting their ability to complete the record prospectively instead of relying on memory recall during their clinic visits. A previous assessment of the validity of maternal reporting of acute healthcare use for children under three found that mothers had greater recall accuracy when their infant was 2–4 months compared to 30–33 months [3]. Other research suggests that the accuracy of recall is dependent on how recent the event was or the method of data collection [2, 4–6]. Sociodemographic characteristics, including maternal age, education, and income, have shown inconsistent effects on recall accuracy [2, 3, 6, 7]. The decline in the completeness of airway-related illnesses recordings with increasing infant age indicates that continued primary physician verification of any illness in infants under 12 months is required. Additional parental support such as reminders, more accessible recording methods, and increasing confidence in classifying airway-related illnesses are needed to prevent systematic underreporting and have been incorporated into the study-specific data collection app for use in the full-scale RCT.

There is limited research on the impact of the introduction of solid foods on the GIT microbiome and the development of the host immune system during complementary feeding. Most of the current evidence has come from cohort or cross-sectional studies, which have only described the association of dietary intake with microbiome diversity and are limited by design. The introduction of solids is a significant event in the first year of life. Understanding the GIT-host microbial dynamics through controlled feeding studies will assist us in delineating the types and composition of foods to optimise the transition from milk feeding (breast and/or formula) to complementary feeding and later to weaning.

## Conclusion

This pilot feasibility study demonstrated that it is possible to recruit infants before introducing complementary foods and achieve good compliance to a food intervention over 6 months without adverse events. The collection of biological samples was primarily impacted by contamination, resulting in changes to sample collection protocols and types of biological samples required to examine the prebiotic food’s longitudinal impact on infants’ microbiome diversity and health outcomes.

## Supplementary Information

**Additional file 1: Fig. S1.** Data collection outline of 40 infants participating in the "Nourish to Flourish" infant complementary feeding pilot feasibility study (2018-2019). **Table S1.** Development of a tree-based analysis for foods consumed by infants participating in the “Nourish to Flourish” infant complementary feeding pilot feasibility study (2018-2019).

## Data Availability

The datasets supporting the conclusions of this article are included within the article (and its additional file(s)).

## Abbreviations

BCR: B cell receptor
CF1: Complementary feeding baseline
CF3: Complementary feeding month 3
CF6: Complementary feeding month 6
GIT: Gastrointestinal tract
HDEC: Health and Disability Ethics Committee
NHI: National Health Index number
NZ: New Zealand
OTUs: Operational taxonomic units
PBMC: Peripheral blood mononuclear cells
RCT: Randomised controlled trial
RS: Resistant starch
SGA: Small for gestational age
URTI: Upper respiratory tract infection
