# Supplementary material for: “Nourish to Flourish”: complementary feeding for a healthy infant gut microbiome—a non-randomised pilot feasibility study
Source: Pilot Feasibility Stud. 2022 May 18;8:103. doi: 10.1186/s40814-022-01059-3 (PMC9116017; doi:10.1186/s40814-022-01059-3)
Supplement: Supplementary file 1 — Additional file 1: Fig. S1. Data collection outline of 40 infants participating in the "Nourish to Flourish" infant complementary feeding pilot feasibility study (2018-2019). Table S1. Development of a tree-based analysis for foods consumed by infants participating in the “Nourish to Flourish” infant complementary feeding pilot feasibility study (2018-2019). [file 40814_2022_1059_MOESM1_ESM.docx]

Supplementary Material

**Inclusion criteria (N=123)**

Healthy infants aged 2 to 3 months

**Baseline assessment (Complementary Feeding 1: CF1)**

*Infant age: ≤ 6 months*

- Questionnaires Samples:
- Anthropometry: length, weight - Mother: breast milk

- Infant: stool, saliva, urine, blood

**Exclusion criteria**

- Born < 32 weeks gestation
- Small for gestational age
- Developmental disability
- Health impairment like to influence nutritional status (e.g. cerebral palsy, malabsorption due to chronic illness)
- A digestive disorder
- Undergoing treatment with an antibiotic
- Receiving a supplement with a pre-pro-biotic
- Parents with written or spoken English comprehension likely to make participation difficult

**Group 1**

*Probiotic control*

n = 10

**Group 2**

*Kūmara powder*

n = 30

**Allocation (N=40)**

**Baseline assessment (Complementary Feeding Month 3: CF3)**

*Infant age: ≤ 9 months*

- Questionnaires Samples:
- Anthropometry: length, weight - Mother: breast milk

- 3 day food record - Infant: stool, saliva, urine, blood

**Baseline assessment (Complementary Feeding Month 6: CF6)**

*Infant age: ≤ 12 months*

- Questionnaires Samples:
- Anthropometry: length, weight - Mother: breast milk

- 3 day food record - Infant: stool, saliva, urine, blood

**Supplementary Figure 1.** Data collection outline of 40 infants participating in the "Nourish to Flourish" infant complementary feeding pilot feasibility study (2018-2019).

**Supplementary Table 1.** Development of a tree-based analysis for foods consumed by infants participating in the “Nourish to Flourish” infant complementary feeding pilot feasibility study (2018-2019).

| Level_1 | Level_1 food group | Level_2 | Level_2 food(s) |
| --- | --- | --- | --- |
| 1 | Breast milk | 1a | Human milk |
| 2 | Formula | 2a | Bovine formula |
|  |  | 2b | Non-bovine formula |
| 3 | Dairy | 3a | Cheeses |
|  |  | 3b | Curdled milk products |
|  |  | 3c | Fermented dairy |
|  |  | 3d | Yoghurt |
|  |  | 3e | Non-bovine milk/products |
| 4 | Meats | 4a | Beef |
|  |  | 4b | Pork |
|  |  | 4c | Lamb, venison, game |
|  |  | 4e | Fish |
| 5 | Eggs | 5a | Eggs |
|  |  | 5b | Egg mixtures |
| 6 | Legumes | 6a | Beans, peas, lentils, soy products |
|  |  | 6b | Nuts, nut butters, and pulses |
|  |  | 6c | Seeds and seed mixtures |
| 7 | High fibre grains | 7a | Wholegrain pastas and bread, brown rice |
|  |  | 7b | Oats and porridge |
|  |  | 7c | Pastas and rice (white) |
|  |  | 7d | White breads |
|  |  | 7e | Infant cereals |
| 8 | Low fibre grains | 8a | Cakes, cookies, pastries, bars |
|  |  | 8b | Crackers and salty snacks |
|  |  | 8c | Pancakes, waffles, French toast |
| 9 | Fruits | 9a | Citrus |
|  |  | 9b | Berries |
|  |  | 9c | Bananas |
|  |  | 9d | Avocados |
|  |  | 9e | Dried fruits |
|  |  | 9f | Tree and stone fruits |
| 10 | Commercial infant foods | 10a | Fruit |
|  |  | 10b | Fruit + yoghurt |
|  |  | 10c | Fruit + grain |
|  |  | 10d | Fruit + veg |
|  |  | 10e | Vege |
|  |  | 10f | Vege + meat |
|  |  | 10g | Vege + meat + grain |
|  |  | 10h | Legumes + dairy |
|  |  | 10i | Fruit + meat + grain |
|  |  | 10j | Vege + dairy |
| 11 | Vegetables | 11a | White potatoes and starchy vegetables |
|  |  | 11b | Dark green vegetables |
|  |  | 11c | Deep yellow vegetables |
|  |  | 11d | Nightshades |
|  |  | 11e | Kūmara |
|  |  | 11f | Other vegetables |
|  |  | 11g | Alliums |
|  |  | 11h | Green beans and peas |
| 12 | Fungi, algae’s, sea veg | 12a | Mushrooms |
|  |  | 12b | Seaweeds |
|  |  | 12c | Algae’s |
| 13 | Fats | 13a | Animal fats |
|  |  | 13b | Plant oils |
|  |  | 13c | Butter |
| 14 | Water | 14a | Water |
| 15 | Sweets and beverages | 15a | Electrolyte drinks |
|  |  | 15b | Lollies |
| 16 | Supplements and Fortifications | 16a | Prebiotics (GOS, FOS, inulin, polydextrose) |
|  |  | 16b | Probiotics |
|  |  | 16c | Vitamin fortification in cereals |
| 17 | Condiments and ingredients | 17a | Salty spreads |
|  |  | 17b | Sweet spreads |
|  |  | 17c | Miscellaneous |
| 18 | Intervention | 18a | Kūmara powder |
| 19 | Imitation and substitution foods | 19a | Nut and soy milks |
|  |  | 19b | Non-dairy yoghurts |
|  |  | 19c | Plant-based meat substitutes |
|  |  | 19d | Fungi derived substitutes (e.g., Quorn) |
|  |  | 19e | Vegan cheese |
| 20 | Herbs and spices | 20a | Herbs |
|  |  | 20b | Spices |
|  |  | 20c | Flavourings (e.g., vanilla and cocoa) |
| 21 | Pre-packaged commercial meals | 21a | Vegetable base |
